# Two-Stage Microwave Hyperthermia Using Magnetic Nanoparticles for Optimal Chemotherapy Activation in Liver Cancer: Concept and Preliminary Tests on Wistar Rat Model

**DOI:** 10.3390/cancers18020330

**Published:** 2026-01-21

**Authors:** Oliver Daniel Schreiner, Thomas Gabriel Schreiner, Lucian Miron, Romeo Cristian Ciobanu

**Affiliations:** 1 Department of Medical Specialties III, Faculty of Medicine, “Grigore T. Popa” University of Medicine and Pharmacy, 700115 Iasi, Romania; schreiner.oliver-daniel@d.umfiasi.ro (O.D.S.); lucian.miron@umfiasi.ro (L.M.); 2 Medical Oncology Department, Regional Institute of Oncology, 700483 Iasi, Romania; 3 Department of Electrical Measurements and Materials, Gheorghe Asachi Technical University, 700050 Iasi, Romania; 4 First Neurology Clinic, “N. Oblu” Clinical Emergency Hospital, 700309 Iasi, Romania

**Keywords:** two-stage microwave hyperthermia, magnetic nanoparticle, liver cancer, Wistar rat, IL-6, TNF-α

## Abstract

**Background/Objectives**: Liver cancer is among the most frequent poor-prognosis malignancies worldwide, with currently insufficient effective treatment. The two-stage microwave hyperthermia using magnetic nanoparticles is a modern technique designed to specifically target tumor tissues and facilitate chemotherapy activation, with promising results from fundamental studies across various tumor types. The method consists of a first irradiation, performed before nano-assemblies administration. This is intended to sensitize the tumor by inducing a hyperthermic effect, leading to increasing blood supply, enhancing endothelial damage/permeation and inflammatory activation, with the final goal of improving the diffusion/retention of nano-assemblies in the tumor. Subsequently, the second microwave irradiation follows the injection in the hepatic artery and diffusion in the tumor of the activated nano-assemblies, to further determine a strong, but localized and focalized hyperthermic action. Nano-magnetic assemblies for hyperthermia accomplish the proposed chemo-thermal delivery, i.e., act per se on the tumor and also destabilize co-administered assemblies of nanoparticles loaded with chemotherapeutics, which would be consequently released locally in the most efficient way. This article aims to demonstrate the efficacy of this therapeutic approach in a rat liver model and its potential applicability in patients with liver tumors. **Methods**: Adult male Wistar rats were used to obtain liver samples, which were divided into three groups, each receiving a different hyperthermia protocol in terms of temperature (41–45 °C), duration, and co-administration of nanoparticles. **Results**: The most suitable exposure temperature for rat liver appears to be 42 °C, resulting in vacuolar degeneration lesions at the focal level. The effects of thermal conditioning do not appear to be homogeneous in the tested liver, and the controlling environment and methodology should be improved in the near future. The level of hepatic inflammation, as indicated by elevated interleukin 6 (IL-6) and tumor necrosis factor alpha (TNF-α) levels, appears negligible under the current hyperthermia protocol. **Conclusions**: Two-stage microwave hyperthermia using magnetic nanoparticles is a promising therapeutic modality for liver cancer, with promising results from animal studies opening the way for further research in humans.

## Simple Summary

Liver cancer has a poor prognosis and limited effective treatments. This study explores a modern therapeutic strategy combining two-stage microwave hyperthermia with magnetic nanoparticles, tested in an adult male Wistar rat liver model. The approach involves an initial microwave heating of the tumor area to increase blood flow and tissue permeability, improving the accumulation of nanoparticles. After nanoparticle administration, a second microwave treatment produces localized heating that directly damages tumor tissue and promotes the local release of chemotherapy drugs carried by the nanoparticles. Our results indicate that 42 °C is the most suitable temperature for liver tissue, inducing localized cellular alterations without causing significant inflammation. Although the hyperthermic effects were not fully uniform, these findings support the potential of this combined chemo-thermal approach. Overall, the described technique represents a promising strategy that warrants further investigation for future application in patients with liver cancer.

## 1. Introduction

Liver cancer remains among the most significant oncological challenges worldwide, both in terms of incidence and mortality [1]. According to the most recent epidemiological reports, hepatocellular carcinoma (HCC), the predominant form of primary liver cancer, ranks as the sixth most common cancer worldwide and the third leading cause of cancer-related death [2]. Additionally, HCC has imposed a significant economic and quality-of-life (QoL) burden on patients, according to recently published systematic reviews [3]. This entity is characterized by poor overall prognosis, largely attributable to late-stage diagnosis, high rates of recurrence, limited responsiveness to systemic treatments, and frequent comorbidities that complicate therapeutic decision-making [4]. The five-year survival rate for advanced liver cancer remains disappointingly low, despite notable advances in surgical techniques, systemic chemotherapy, targeted agents, and immunotherapy [5]. This enduring clinical burden underscores the urgent need for novel, effective treatment modalities that selectively target tumor tissue while sparing surrounding healthy structures.

Conventional therapeutic strategies for liver and gastric malignancies have well-recognized limitations. For HCC, curative resection or liver transplantation is often precluded by underlying cirrhosis, multifocal disease, or insufficient donor availability [6]. Ablative approaches such as radiofrequency ablation (RFA) [7] or transarterial chemoembolization (TACE) [8] are associated with incomplete necrosis in larger lesions and high recurrence rates. Systemic therapies, including tyrosine kinase inhibitors [9] and immune checkpoint inhibitors [10], have yielded modest improvements in survival, but drug resistance, systemic toxicities, and high treatment costs constrain their efficacy. First- and second-line chemotherapeutic regimens, combined with targeted therapies such as trastuzumab in HER2-positive tumors or immune checkpoint inhibitors, have expanded the therapeutic alternatives. Still, the median survival of patients with advanced gastric cancer continues to be less than 12 months [11].

Hyperthermia therapy has reemerged as a promising modality in oncology by elevating tumor tissue temperatures to 41–45 °C, thereby inducing direct cytotoxic effects, disrupting tumor microenvironments, and enhancing the efficacy of radiotherapy and chemotherapy [12]. Tumor cells, more heat-sensitive than adjacent normal tissues, undergo impaired deoxyribonucleic acid (DNA) repair, apoptosis, and necrosis under hyperthermic stress [13]. However, the clinical application of conventional hyperthermia techniques, such as radiofrequency, ultrasound, or whole-body heating, has been hindered by difficulties in achieving precise, localized, and sustained heating in deep-seated tumors, like those of the liver and stomach [14]. Nanotechnology has provided innovative solutions to these challenges, particularly for magnetic nanoparticles (MNPs), such as iron oxide–based MNPs, which can preferentially accumulate in tumors via enhanced permeability and retention [15,16,17,18]. Such particles can be activated by external energy sources, as reported in the literature [19,20]. Preclinical studies in several tumor models have already shown that a simple hyperthermia in the presence of MNPs achieves uniform heating, tumor necrosis, and immune stimulation, positioning it as a compelling candidate for treating intra-abdominal malignancies such as liver and gastric cancer [21].

Still, there is a compelling rationale for exploring alternative strategies that can provide local tumor control, enhance the efficacy of systemic treatment, and potentially improve long-term outcomes. In this paper, a two-stage microwave hyperthermia protocol is proposed, with two stages following each other as follows: 1. Preliminary hyperthermia to activate the tumor cells; 2. Injection of cytostatic assemblies based on magnetic nanoparticles, which attach with priority to the pre-activated tumor cells, followed by a second stage hyperthermia, this time dedicated to breaking the nano-assemblies to liberate the cytostatic exactly to the tumor cell, in terms of precision medicine. The second stage of hyperthermia exhibits a synergistic effect: beyond local administration of the cytostatic, MNPs stimulated by microwave irradiation generate additional localized heat within tumor cells, thereby increasing their sensitivity to the treatment. This strategy clearly provides superior spatial control, deeper tissue penetration, and the potential for repeat treatments without systemic toxicity.

Despite these promising findings, a critical need remains to demonstrate the translational potential of two-stage microwave hyperthermia with magnetic nanoparticles in relevant tumor models that closely mimic human disease. Preclinical studies are essential not only to confirm the safety and efficacy of this modality but also to help elucidate the mechanisms of action, optimize nanoparticle delivery and distribution, and evaluate its potential integration with existing treatment regimens. The liver is a favorable starting point for such investigations, given its high incidence of primary and metastatic malignancies, its anatomical accessibility for local nanoparticle delivery, and the well-established limitations of current therapies.

In this context, the present study aims to: first, evaluate the efficacy of two-stage microwave hyperthermia using magnetic nanoparticles in a rat model of liver cancer, with particular attention to tumor control, histopathological changes, and potential effects on surrounding hepatic parenchyma; and second, to illustrate the translational applicability of this therapeutic principle in humans. The ultimate goal is to establish a proof-of-concept framework for nanoparticle-mediated hyperthermia as a viable treatment strategy for solid tumors with a poor prognosis, serving as a foundation for further translational research and ultimately clinical trials aimed at improving outcomes for patients with liver cancer.

## 2. Materials and Methods

### 2.1. Animal Model

For the experiments, adult male Wistar rats (obtained from the Victor Babeş National Institute for Research and Development, Bucharest, Romania) weighing 180–200 g at the beginning of the experiments were used. A total of 18 rats were used, 6 animals/study group. Males were preferred for several reasons: to reduce biological variability, to achieve more stable hepatic enzyme expression and pharmacokinetics, and to exhibit better tolerance to microwave hyperthermia. The animals are housed in a temperature-controlled room (21 °C ± 2 °C) with a 12 h light/12 h dark circadian rhythm, with one rat per cage, and are allowed to acclimate to this environment for at least 24 h before the experiment. They have free access to food and water. The cages are standardized polycarbonate (PACO) cages, measuring 1500 cm^2^.

### 2.2. Euthanasia of the Animals

At the end of the experiment, the animals are euthanized by causing a rapid death, without physical and mental suffering, according to the AVMA Euthanasia Protocol of 2000 (the method must be painless, rapidly produce unconsciousness, cardiac arrest, respiratory arrest, and death) [22]. This is a standard procedure and is performed in specially equipped necropsy rooms, separate from the area where other animals are housed.

The euthanasia procedure that was chosen consists of the inhalation administration of a volatile anesthetic (enflurane or isoflurane), which induces unconsciousness within a few seconds. The absence of vital signs (heartbeat, respiratory movements, reflexes) is monitored for a period of over 5 min. After confirmation of death, tissue samples are collected.

### 2.3. Tissue Preparation

To achieve this stage, a closed system was required to ensure the recirculation of a fluid that could be controlled and maintained at a specific temperature for a predetermined period. This type of system was chosen because it provides optimal control to maintain the required temperature for constant heat transfer among the system components. The prototype made within the Biomedical Research Center (Grigore T. Popa University of Medicine and Pharmacy Iasi, Romania) consists of the following elements: water bath, water recirculation pump, water thermometer, thermostat, paddles, water bath-paddle connection tubing, external body heating system, and cold light source.

The paddles were explicitly designed for this experiment using polyethylene tubes with an outer diameter of 1.27 mm and an inner diameter of 0.86 mm. These tubes were arranged in a circular pattern on a self-adhesive platform, forming two flexible paddles with a diameter of 20 mm. After securing the tubes to the self-adhesive platform, the next step was to insulate the external side. This was necessary to achieve unidirectional heat transfer to the region between the two paddles, thereby confining the maximum temperature variation to the tissue between the paddles. In contrast, the temperature of the surrounding environment would not be affected. These paddles were connected to the water bath via polyethylene tubes of the same diameter as those above, each 45 cm long, with a tap added to distribute water evenly to the two paddles. It should be noted that there is approximately a 2 °C difference between the water bath and the paddles.

To quantify the temperature at the level of the liver lobe of the experimental animal, an electronic thermometer with an accuracy of 0.1 °C was used, whose flexible transducer with a diameter of approximately 1.5 mm was inserted into the liver before, during heating, and maintained for approximately 2 min after the end of this process. To maintain a constant body temperature, which decreases during laparotomy in rats, an external heating system was used. This was set to 39 °C.

### 2.4. Selective Intrahepatic Transarterial Administration System in Rats

For the selective injection of nanoparticles into the liver, the following procedural technique was used:

- Anesthetizing the animal with a mixture of ketamine 65 mg/kg and xylazine 15 mg/kg;
- Disinfection of the abdominal and inguinal skin with betadine;
- Making an incision at the inguinal level, followed by dissection of the subcutaneous cellular tissue until the vascular-nerve plane is released, with identification of its elements;
- Individualization with maximum protection of the femoral artery (0.9 mm diameter), which will be exposed using two guide wires;
- Catheterization of the femoral artery with a polyethylene tube with an outer diameter of 0.61 mm and an inner diameter of 0.28 mm;
- Insertion of the catheter up to the level of the abdominal aorta.

The next stage consists of performing the Mercedes laparotomy to provide good exposure of the internal organs.

Because of the rat’s particular anatomical configuration, the next step was to expose the entire abdominal aorta. After locating it and the emergence of the celiac trunk, dissection was arranged at this level with the lifting of a segment of the aorta and the celiac trunk from the posterior plane.

The catheter introduced at the femoral level is directed through the aorta into the celiac trunk, where it will later be fixed by ligature.

**Figure 1:**
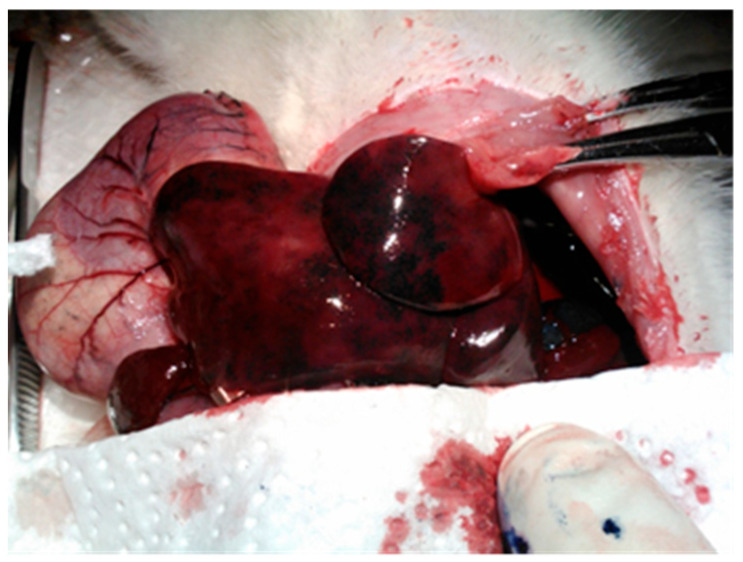
Intraoperative view of the liver tissue in rats.

For the selective administration of various substances to the liver, it is necessary to individualize and clamp all the collaterals emerging from the celiac trunk, except for the hepatic artery. Figure 1 shows an intraoperative view of the liver tissue obtained for further experiments.

### 2.5. Sample Allocation

The experiments were performed in duplicate or quadruplicate for each condition required by the experimental design. The first protocols required trial and error to establish optimal experimental conditions, and the number of experimental animals used was higher, including those that failed and had to be repeated. After prior anesthesia with ketamine and xylazine, the experimental animal was placed in the decubitus position on the external heating platform and subsequently underwent a Mercedes laparotomy for good exposure of the peritoneal cavity. After careful isolation of the operating field, the hepato-diaphragmatic and hepato-gastric ligaments were released, thus resulting in appropriate mobilization of the three hepatic lobes. The lower lobe was chosen for liver heating, and the other two served as control lobes. To achieve hyperthermia in the liver, the lower lobe was placed between the two heated paddles, and to monitor liver temperature, a thermometer probe was inserted at this level. After reaching the desired temperature at the level of the liver lobe, it was removed from the two paddles and allowed to slowly return to the initial temperature.

To determine the influence of different parameters (exposure temperature, harvesting interval) and the impact of nanoparticles on the structures of the liver parenchyma, experiments were performed on three distinct groups of animals or tissues, as follows:

- The first batch included rats whose liver lobes were heated at 42 °C before MNPs;
- The second batch included rats whose liver lobes were heated at 42 °C, and subsequently nanoparticles were administered;
- The third batch studied rats whose liver lobes were heated at 45 °C, and subsequently, nanoparticles were administered.

### 2.6. Microscopy as the Main Technique for Tissue and Nanoparticle Examination

A significant step in evaluating the pharmacodynamics of nanostructures used in the experiment involves directly or indirectly identifying nanoparticles in the tissues they reach, using alternative loading methods with substances that enable image development.

The preparations for microscopy were of three categories: excision pieces fixed in 10% buffered formalin, embedded in paraffin (with processing according to the standard morphopathology protocol, including incubations in ethanol, isopropanol/amyl alcohol, paraffin at 56 °C, xylene), fingerprints, and deposits (on glass slides) of nanoparticle powder loaded with Nile red, a fluorochrome visible in direct optical microscopy or confocal microscopy, the substance assumed to be stable.

In most cases, multiple samples were placed in the same embedding cup (to facilitate comparisons of microscopy images across different organs or donor subjects, and to standardize the effects of histological processing). Microscopic sections were generated at 4 µm on standard 24 × 75 mm slides; up to 6 were produced per embedding block, either individually on separate slides or in bulk on the same slide (to minimize processing variation). Organ section impressions (liver) were received in multiple layers on the same slide, with air-drying fixation.

Microscopic examinations were performed in the following three variants:

- No staining;
- Standard Hematoxylin staining, for nuclei;
- Standard Hematoxylin-Eosin staining (for morphology); this serves to identify interferences in fluorescence between pigments (endogenous/marking) and the fluorochrome used for nanoparticles (Nile red).

The immediate analytical destination of these materials includes three methods (standard transillumination evaluation, fluorescence evaluation, and fluorescence colocalization). Three microscopes are combined for exploration: Nikon Eclipse E800 (Nikon, Minato City, Tokyo, Japan), for rapid evaluations, Zeiss Observer (Karl Zeiss AG, Oberkochen, Germany), including Z1—for constructing digitized images of scanning preparations and Z2—for fluorescence detailing at resolution and, subsequently, colocalization. The imaging software in the last two versions is incompatible, necessitating separate maintenance of evidence for the localization of elements within the section space.

Regarding fluorescence microscopy, we used a confocal microscope with Laser scanning Leica direct microscope DM 5500Q (Leica Camera AG, Wetzlar, Germany) with Leica TCS SPE confocal equipment and accessories (camera), and for data processing, a LAS AF software, version Lite 4.0 (Leica Camera AG, Wetzlar, Germany) image acquisition and processing system-Leica DFC 290 camera and adapter with Leica Application Suite LAS software, version 2.8.1 (Leica Camera AG, Wetzlar, Germany). The preparations for microscopy were of 3 categories:

- Excision pieces (rat liver) included in GSV1 medium and cryofixed with tetrafluoroethane—containing nanoparticles (RG502H and RGP) loaded with the Nile red fluorochrome;
- Excision pieces (rat liver) fixed in 10% buffered formalin, embedded in paraffin (with processing according to the standard morphopathology protocol, including incubations in ethanol, isopropanol/amyl alcohol, paraffin at 56 °C, xylene)—containing nanoparticles loaded with magnetite (Fe_3_O_4_) and stained with Prussian blue (Perls stain);
- Deposits (on glass slides) of nanopowder loaded with Nile red, assumed to be stable (the slide-to-slide adhesion solutions used were either DPX or distilled water).

Cryofixed microscopic sections were generated at 5–10 microns on standard 24 × 75 mm slides, produced up to 24 per embedding block, grouped either distinctly on slides or in bulk on the same slide (to minimize processing variations). Paraffin-embedded microscopic sections were cut at 4 µm on standard 24 × 75 mm slides; up to 6 sections were produced per inclusion block, either individually on separate slides or in bulk on the same slide (to minimize processing variation).

Microscopic examinations were performed in the following two variants:

- Without staining—for fluorescence microscopy observation of the fluorochrome used for nanoparticles (Nile red);
- Standard Hematoxylin-Eosin staining (for morphology) and Prussian blue staining (Perls staining) for Fe_3_O_4_ examination.

The samples to be studied were examined under a microscope using fluorescence, differential phase contrast, polarized light, bright-field, and confocal illumination.

### 2.7. Design, Development, and Testing of Magnetic Nanoparticles

Ultrafine, uniform, approximately spherical, and high-purity Fe_3_O_4_ nano-powders, with a size of 8–20 nm, were prepared by the controlled chemical co-precipitation method from ferrous/ferric solution by mixing with saline solution in an alkaline medium. Tests were performed to highlight the effects of temperature, solution pH, stirring rate, and oleic acid concentration on the size of Fe_3_O_4_ nanoparticles. It is well established in the literature that Fe_3_O_4_ nanopowders exhibit good biocompatibility [23,24,25]. In our case, they can be easily dispersed in aqueous solutions due to the presence of COO- on the surface of the nano-powders. Ferrite nano-powders with oleic acid (OA-Fe_3_O_4_) were prepared by chemical co-precipitation of Fe (III) and Fe (II) cations in a 1:2 molar ratio under dilute basic conditions (NH_4_OH). Oleic acid, C_18_H_34_O_2_, is an unsaturated carboxylic acid with a double bond in the middle of its chain. The node plays a crucial role in organizing surfactant (stabilizer) molecules on the surface of magnetic particles, thereby enabling proper steric repulsion. It is considered one of the most effective stabilizing agents for ferrite nanopowders in organic solvents, owing to the polar heads’ chemisorption on the surface of magnetic particles. At the same time, the hydrophobic tails dissolve in the dispersion medium.

**Figure 2:**
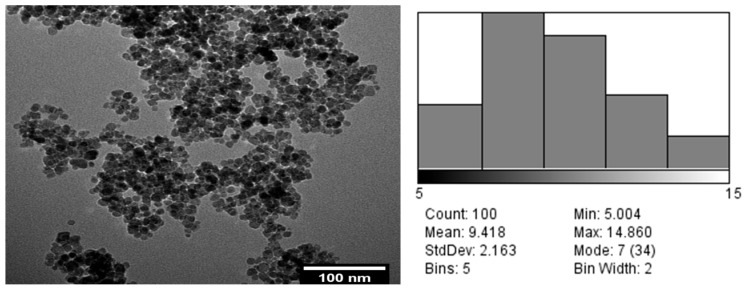
Morphology of the magnetite particles studied by TEM (**left**) and average particle size (**right**).

The morphology of the magnetite particles was studied by TEM (Figure 2), and the average particle size and particle-size distribution were estimated by performing at least 100 measurements using a dedicated software program. From analysis of the TEM micrograph and the particle size distribution, magnetite nanoparticles with a regular, almost spherical shape, a relatively high degree of agglomeration, and sizes ranging from 5 to 15 nm, with an average size of 9.418 nm, were obtained.

Dielectric tests were performed over the temperature range of 25 °C to 50 °C and the frequency range of 1 Hz to 10^9^ Hz using the Broadband Dielectric Spectrometer (Novocontrol GmbH, Montabaur, Germany), which comprises an alpha frequency response analyzer and a Quattro temperature controller, with tailored measurement cells.

The nanoparticle type OA-Fe_3_O_4_ (100 µL) mixture exhibits high stability with respect to temperature and frequency, maintaining its properties up to 50 °C. Beyond this temperature, the dielectric constant decreases by approximately 2% with increasing frequency. The optimum temperature of OA-Fe_3_O_4__100 µL is 40–45 °C. In the case of OA-Fe_3_O_4__200 µL, the dielectric constant is higher due to the higher ferrite concentration and greater stability at elevated temperatures, with an optimal temperature of 45–50 °C [26].

Prior to the microwave hyperthermia experiments, toxicity tests were conducted both in vitro and in vivo. The in vitro test evaluated liver cell viability using the MTT (3-(4,5-dimethylthiazol-2-yl)-2,5-diphenyltetrazolium) assay. Once the treatment period ended, the medium was removed, the cells were rinsed with PBS, and 100 µL of fresh complete medium was added. Subsequently, 10 µL of MTT (5 mg/mL) was incorporated into the medium, and the cells were incubated for 3 h. DMSO (dimethyl sulfoxide) was used to dissolve formazan generated by the metabolism of living cells, and absorbance was measured at 570 nm using a plate-reading spectrophotometer. Cell viability (%) was determined using this formula: % cell viability = sample [absorbance]/control [absorbance] × 100. The tests were conducted three times, yielding consistent results.

Following the in vitro test results, in vivo experiments were conducted in the animal model. The agent was administered as a single dose; the evaluation of nanoparticle biocompatibility in the animal body involved assessing acute systemic toxicity. Testing conditions before and after administration were in accordance with international guidelines, with dosage, time of administration, and other relevant parameters recorded for each animal. Analysis of the health status of the experimental animals 24 h after nanoparticle administration, relative to the control group, revealed no significant alterations, indicating excellent biocompatibility.

### 2.8. Aspects Related to Electromagnetic Simulation of Liver Tissue and Exposure to the Microwave Domain for Hyperthermia Effects Evaluation

Extensive research by [27,28,29,30] established the dielectric relaxation model of tissues, usable in CST Studio Suite [31], as a basis for developing electromagnetic models of the human body. Ongoing research aims to determine the variation in certain electromagnetic parameters with age, patient’s sex, and other variables [32]. The simulation followed the methodology described in detail by [33,34,35]. The methods for applying microwave energy to the targeted liver tissue were as described in [36,37,38], using specialized antenna-electrodes inserted directly into the tumor. Finally, the exposure mechanism was evaluated under 2.45 GHz microwaves at room temperature, and the evolution of liver tissue temperature was monitored during the two stages of the hyperthermia protocol. Liver tissue temperature was monitored invasively, as detailed in [39], although non-invasive methods are also possible, as reported in the literature [40,41].

Given the methodology of guiding microwave energy through specialized antenna electrodes directly implanted in the liver, the impact on surrounding organs was deemed negligible. But the potential for radiation to penetrate toward the kidneys, which are located near the treated area of the liver, was also considered. The results were included in the paper, related to the highest level of accumulated radiation.

### 2.9. Qualitative Determination of IL-6 and TNF-α by RT-PCR Technique

Reverse transcription polymerase chain reaction (RT-PCR) was used to quantify interleukin-6 (IL-6) and tumor necrosis factor-α (TNF-α) expression in rat liver homogenate. IL-6 is an interleukin with both pro-inflammatory and anti-inflammatory properties, secreted by T cells and macrophages to stimulate the immune response (post-inflammation or post-trauma). TNF-α is a pro-inflammatory cytokine whose name derives from its ability to destroy tumor cells and induce hemorrhagic necrosis in tumors transplanted into mice. TNF-α is produced by several types of cells, including macrophages, monocytes, lymphocytes, mast cells, neutrophils, keratinocytes, astrocytes, microglia, smooth muscle cells, and some tumor cell lines. Large amounts of TNF-α are released upon contact of macrophages, CD4+ T lymphocytes, and natural killer (NK) cells with lipopolysaccharides, bacterial products, and interleukin 1 (IL-1). The exact multi-step protocol of RT-PCR is presented in detail in several previous works [42].

### 2.10. Ethical Consent

The study began after receiving a favorable opinion from the Ethics Committee of ‘Grigore T. Popa’ University of Medicine and Pharmacy Iasi, Romania (University ethical approval number: 50/17 January 2021) and of the National Veterinary Agency (DSVSA) of Romania (ethical approval number: 33/7 April 2021). The study was conducted in accordance with the recommendations of the European Community regarding the conduct of preclinical studies with medicinal products (European Community Council Directive 2010_63_EU) and in accordance with the Principles of Good Laboratory Practice, as well as the inspection and verification of their compliance in the case of testing carried out on chemical substances, published in the Official Gazette, Part I no. 102 of 2 June 2002 (approved by the Decision of The Government of Romania no. 63 of 24 January 2002) as well as in accordance with the Analytical, Pharmacotoxicological and Clinical Norms and Protocols regarding the testing of medicinal products (approved by the Order of the Ministry of Public Health 906 of 25 July 2006).

## 3. Results

### 3.1. Simulation of Exposure of Liver Tissue to the Microwave Domain for Hyperthermia Effects Evaluation

**Figure 3:**
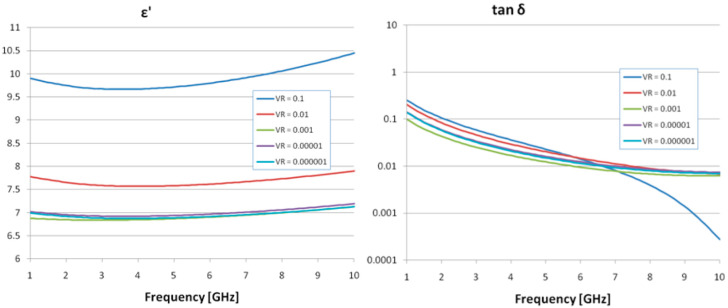
Dielectric properties of the samples with Fe_3_O_4_ composite inserts.

The results of the dielectric evaluation (dielectric permittivity, ε, and loss factor, tan δ) are presented in Figure 3 for various volume ratios (VR) of OA-Fe_3_O_4_ inserts across the GHz frequency range. The dielectric permittivity exhibits a gradual increase with frequency, but the number of inserts significantly affects the values. Concerning the Tan δ characteristic, a reduction in the characteristic was observed with increasing frequency, and the effect of insert content is minimal. Ultimately, it was determined that a 0.1% OA-Fe_3_O_4_ insert concentration improves the dielectric properties, and the selected frequency of 2.45 GHz for the hyperthermal tests is appropriate when considering the dielectric parameter values.

**Figure 4:**
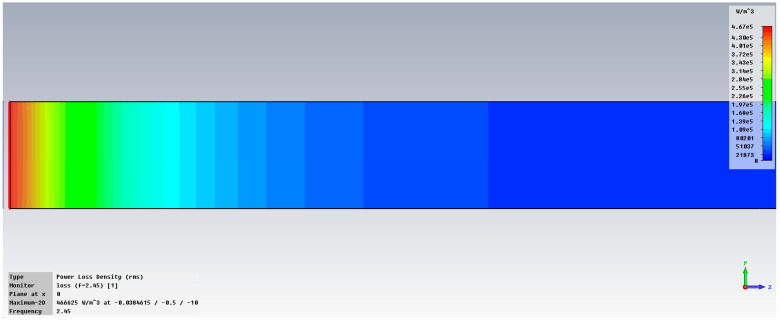

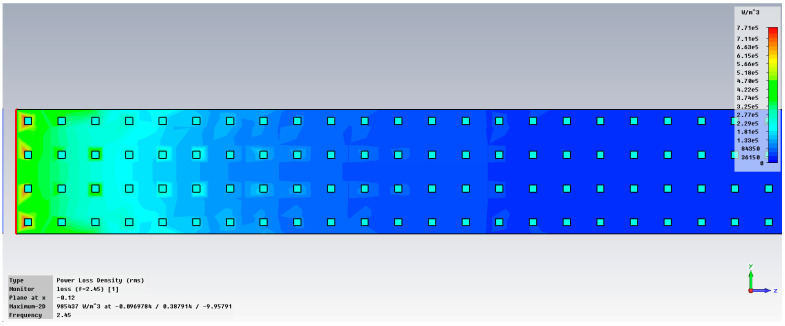
Variation in dissipated energy at 2.45 GHz in liver tissue, without inserts (**up**) and with 0.1% OA-Fe_3_O_4_ inserts (**bottom**).

The synthesis of the simulation results is presented in Figure 4 for the addition of 0.1% OA-Fe_3_O_4_ inserts. The simulation concerns the penetration of the electromagnetic field in a vertical profile (y-z).

The maximum specific absorption rate—SAR is 466,625 W/m^3^ in liver tissue without inserts and, respectively, 985,437 W/m^3^ in liver tissue with OA-Fe_3_O_4_ inserts. It should be noted that an addition of 0.1% OA-Fe_3_O_4_ inserts increases the accumulated energy in liver tissue by 111%. It is also observed that the inserts increase the penetration depth into the tissue and improve the uniformity of energy dissipation within it. This explains the temperature increase after injecting the MMP solution into the hepatic artery and its diffusion into the tumor, which, in turn, results in an enhanced, but localized and focal, hyperthermia effect.

**Figure 5:**
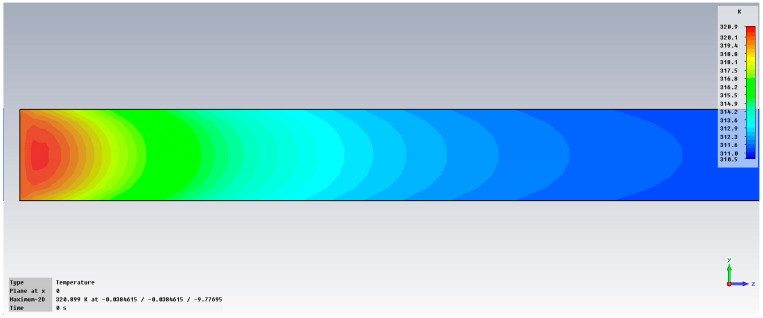
Variation in temperature at 2.45 GHz in liver tissue, with 0.1% OA-Fe_3_O_4_ inserts.

Figure 5 presents the variation in temperature in liver tissue, with inserts, after exposure at 2.45 GHz. It can be observed that the temperature in the area of interest may increase up to 45 °C.

**Figure 6:**
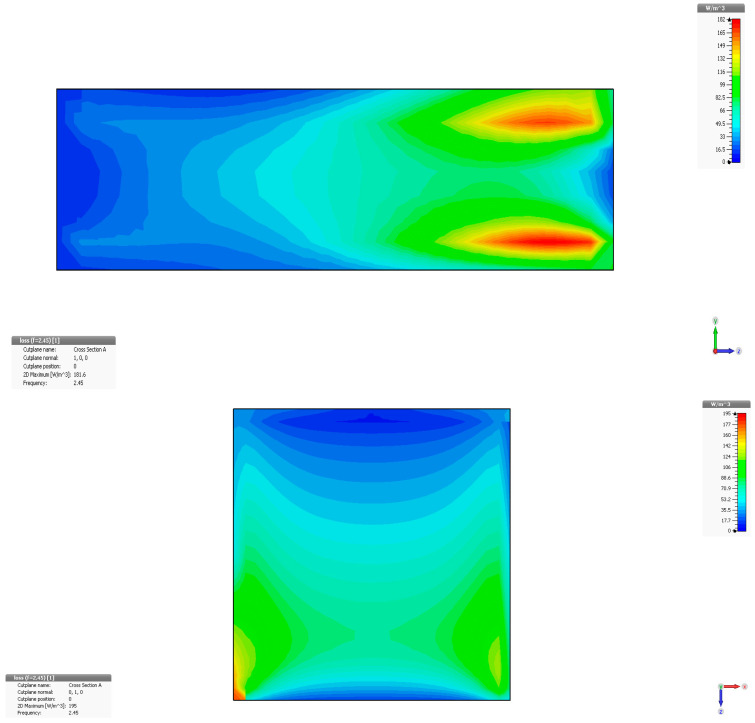
Power loss density in liver tissue, vertical and horizontal planes at 2.45 GHz.

Figure 6 displays the examination of power loss density in liver tissue at 2.45 GHz, shown as a three-dimensional perspective (vertical y-z and horizontal x-z planes), when utilizing two antennas. The method can accurately concentrate energy on the specific region of interest in the liver, specifically the targeted cancerous area.

**Figure 7:**
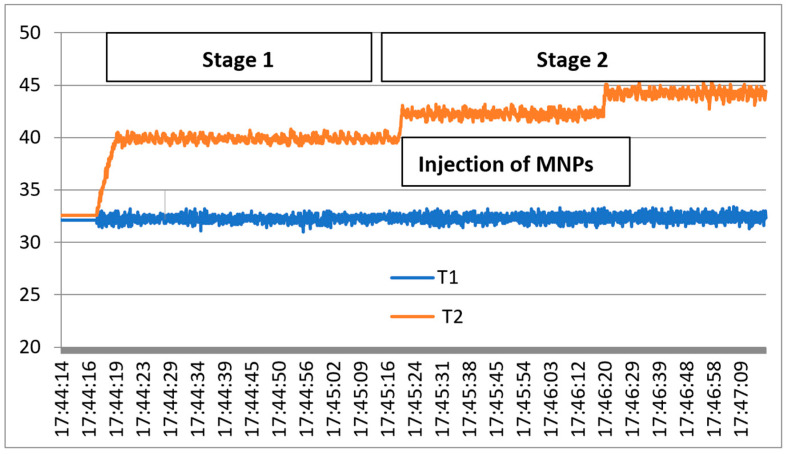
Exposure results under the two stages of microwave hyperthermia.

The exposure mechanism under 2.45 GHz microwaves is presented in Figure 7, where T1 denotes the rat temperature reference and T2 the temperature evolution in liver tissue during the two stages of applied hyperthermia, as described in [39]. Stage 1.—Preliminary hyperthermia to activate the liver cells, up to 40 °C. Stage 2.—Injection of the compound based on 0.1% OA-Fe_3_O_4_ nanoparticles, which attach with priority to the pre-activated tumor cells, followed by second-stage hyperthermia, up to 44 °C.

### 3.2. The Impact of Hyperthermia on the Tissue Samples

The changes observed in the first batch include vascular stasis and congestion around the portobiliary spaces, subcapsular hemorrhage with polymorphonuclear leukocytes (PMN) accumulation, and hydropic degeneration around the centrolobular vein. In another liver preparation under the same conditions, in addition to areas of hepatocyte necrosis, a rich inflammatory infiltrate with PMNs and fibrinolymphocyte deposits was observed on the liver capsule. The subcapsular location and intraparenchymal extension of the areas of hepatocyte necrosis, accompanied by an inflammatory infiltrate with PMNs, could be explained by the friability of the liver induced by the thermal agent. The definitive temperature-induced changes are those manifested as vacuolar degeneration.

**Figure 8:**
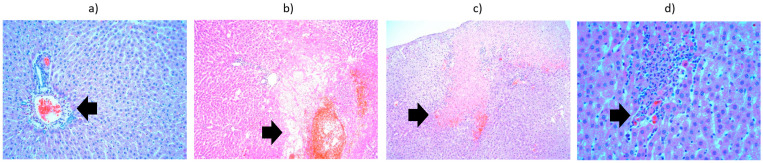
Liver from the third batch, different heating protocols comparison—histopathological aspects highlighted by black arrows: (**a**) at 15 min, showing infiltrate (lymphocytes, plasma cells, eosinophils) in the portobiliary spaces (×10); (**b**) at 24 h, showing area of hepatocyte necrosis and neutrophil infiltrate (×10); (**c**) at 48 h, showing vacuolar degeneration, subcapsular necrosis infiltrated with neutrophils (×10); (**d**) at 9 days, showing lympho-plasmocytic infiltrate, with intraparenchymal neutrophils and unicellular necrosis (×40).

Regarding the results obtained from the first batch, the liver preparation heated to 42 °C and harvested at 15 min showed, in addition to vacuolar degeneration, a lymphoplasmacytic inflammatory infiltrate associated with eosinophils in the portobiliary spaces and small foci of intraparenchymal lymphoplasmacytic inflammatory infiltrate. The liver preparation heated to 42 °C and harvested at 24 h showed areas of intraparenchymal hepatocytic necrosis with PMN infiltration, fibrinolymphocyte deposits on the liver surface, and hydropic degeneration. The liver preparation heated to 42 °C and harvested at 48 h showed vacuolar degeneration and areas of hepatocytic necrosis, with a lymphoplasmacytic inflammatory infiltrate and PMNs located subcapsularly. The liver preparation heated to 42 °C and harvested at 9 days showed significant vacuolar degeneration, small intraparenchymal lymphoplasmacytic aggregates, and, in the portobiliary spaces, eosinophils. Intraparenchymal hepatocytic necrosis accompanied by an inflammatory lympho-plasmacytic infiltrate, associating PMNs and subcapsular PMN agglomerations are noted. It can be concluded that the liver, heated to 42 °C and harvested at 15 min, initially exhibits small intraparenchymal lympho-plasmacytic foci. Consequently, by 24 h, 48 h, and 9 days, areas of hepatocytic necrosis associated with PMN infiltration are fully developed (Figure 7). Hydropic degeneration is a phenomenon present in all preparations regardless of the time at which they were harvested. The fibrin-leukocyte deposits on the liver surface, which initially appear at the 24 h harvest, are also preserved at 48 h and 9 days (Figure 8).

**Figure 9:**
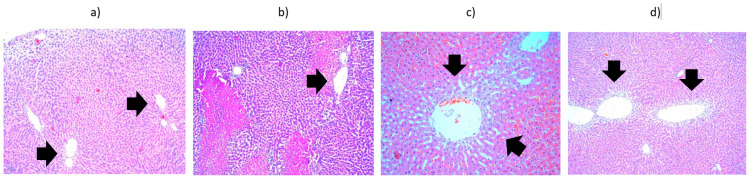
Liver from the fifth batch, different heating protocols comparison—histopathological aspects highlighted by black arrows: (**a**) 1 min at 42 °C, presenting hydropic degeneration (×10); (**b**) 3 min at 43 °C, presenting hydropic degeneration and areas of intraparenchymal hepatocyte necrosis with inflammatory infiltrate (×10); (**c**) 5 min at 42 °C, showing hydropic degeneration around the centrolobular vein (×20); (**d**) heated 10 min at 42 °C, showing vacuolar degeneration (×10).

The second batch, which comprised liver samples heated for 1 min at 42 °C, showed predominantly subcapsular hydropic degeneration. Liver heated for 3 min at 42 °C showed hydropic degeneration around the portobiliary spaces; liver heated for 3 min at 43 °C showed hydropic degeneration and numerous areas of intraparenchymal hepatocyte necrosis with an inflammatory infiltrate containing PMNs. Liver heated for 5 min at 42 °C showed vacuolar degeneration around the centrilobular vein. Liver heated for 10 min at 42 °C showed vacuolar degeneration throughout the hepatic acinus. In conclusion, hydropic degeneration is observed regardless of the duration of heating; only its location varies: subcapsular (liver heated for 1 min), around the portobiliary space (3 min), around the centrilobular vein (5 min), and throughout the acinus (10 min) (Figure 9). The right kidney, heated for 5 min at 42 °C, presented significant interstitial hemorrhages without being able to highlight hydropic degeneration, while the left kidney (control) appeared normal. The right kidney heated for 1 min at 42 °C showed hydropic degeneration in the convoluted tubules. The situation of a control kidney with hydropic degeneration in the absence of thermally induced lesions is also presented. It can be considered that manipulation of the kidneys outside the renal lodges can induce cellular suffering, but also a more significant hypothesis: selective catheterization of the hepatic artery with a catheter, even a very thin one, can induce hypoxic lesions either in the liver or in the kidneys located in the area of passage of the catheter.

**Figure 10:**
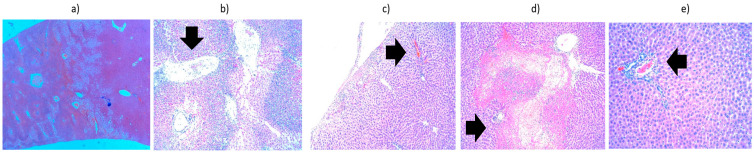
Liver from the third batch, heated to 45 °C—histopathological aspects highlighted by black arrows: (**a**) overview (×2.5); (**b**) extensive area of hepatocyte necrosis, neutrophil exudate, panacinar necrosis (×10); (**c**) subcapsular necrosis and fibrino-leukocyte deposits on the liver surface (×10); (**d**) intraparenchymal hepatocyte necrosis and lympho-plasmacytic inflammatory infiltrate (×10); (**e**) discrete lympho-plasmacytic inflammatory infiltrate in the portobiliary spaces (×20).

The third batch is represented by liver heated to 45 °C (Figure 10), which showed a significant area of hepatocytic necrosis occupying half of the section, accompanied by abundant exudate containing PMNs. The necrotic changes are panacinar: areas 1 and 2 of the hepatic acinus show total necrosis, whereas area 3 shows only significant coagulation changes (intense eosinophilic cytoplasm and hyperchromatic nucleus). The other half of the section presented a discrete inflammatory infiltrate in the portobiliary spaces and hydropic degeneration. Important fibrino-leukocyte deposits were also noted on the surface of the liver.

**Figure 11:**
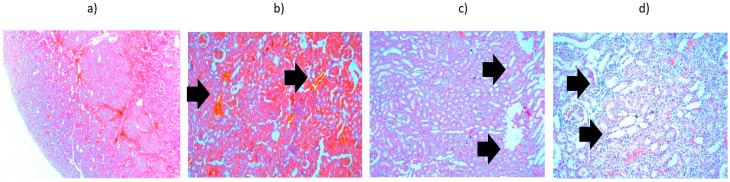
Kidney tissue heated to 42 °C—histopathological aspects highlighted by black arrows: (**a**) overview (×2.5); (**b**) interstitial hemorrhages (×10); (**c**) vacuolar degeneration of convoluted tubules (×10); (**d**) vacuolar degeneration of convoluted tubules (×20).

The potential for radiation to penetrate toward the kidneys, which are located near the treated area of the liver, was also considered. The results shown in Figure 11 pertain to the highest level of accumulated radiation. It was noted that interstitial hemorrhages and vacuolar degeneration of convoluted tubules may occur, but no hydropic degeneration, suggesting minimal side effects on organs near the liver under in vivo conditions.

### 3.3. The Optimal Method for Tissue Preparation and Examination

Across various histological tissue preparations, a clear conclusion is that standard histological processing with paraffin embedding is incompatible with fluorescence-based methods for detecting Nile red-labeled nanoparticles. Tests performed in numerous experiments have shown that, in the alcohol fixation variant, fluorochrome-labeled particles cannot be identified, although they can be detected using standard microscopy methods. We believe that the fluorochrome (liposoluble) is eluted during the process. In contrast, liver fingerprints show us images corresponding to the fluorescence of the target particles.

Thus, the following two complementary methods for generating histological preparations should be used: cryotomy, with the disadvantage of large section thickness, compensated for by examination in colocalization, and with the guarantee of preserving the fluorescence in Nile red, and inclusion in epoxy resins (from the non-hard category—to protect microtome slides). We mention that, in the event of subsequent gene expression analysis (feasible in our country), histological fixation in gentle reagents (RNA-save type) and the abandonment of formalin may be necessary.

Nanoparticles are not homogeneous. The suspensions were evaluated by fluorescence microscopy to assess the concordance between the microscopic features and the distribution reported by the partner who characterized the nanoparticle populations. The images we obtained enable us to describe at least 3-dimensional populations in fluorescence microscopy. An even more precise characterization can be achieved using standard flow cytometry in our country, but this has not yet been performed. The nanoparticle populations are not homogeneous, but the dimensional distribution does not pose significant problems for conducting the experiments. It does not affect tissue fixation results because the dimensions are within the manufacturer’s limits.

The effects of thermoconditioning do not appear homogeneous across the liver mass, nor are they controllable with the methodology used to date. The study was complex, in which we evaluated the distribution of thermal changes in the heated liver mass to quantify lesions within the thermal limits we used. Both on hematoxylin and eosin staining and on fluorescence microscopy, we can identify distortions of varying degrees (ranging from cytoplasmic condensations to cellular fragmentation and secondary inflammatory infiltrates) that are irreversible lesions. We can identify large areas of organs that appear unaffected, as well as neighboring areas with major distortions. Accordingly, we can identify an endothelial effect of heat exposure that can be quantified by imaging: a marked decrease in the number of endothelial cells in small vessels in lesional areas compared with apparently unaffected areas. It is likely that detailed analysis at the level of individual microscopic fields for the entire series of heat-treated animals would be necessary. We recommend adopting a thermal exposure protocol with greater control and incorporating immunohistochemistry (available in our laboratory) into the series of microscopic evaluations to clearly document thermoadaptation (targeting variation in heat shock protein expression) and endothelial function.

Neither Nile red nor magnetite-loaded nanoparticles could be observed under optical microscopy on rat liver section slides. No differences were observed between sections containing Fe_3_O_4_ nanoparticles administered 10 min before or 5–10 min after liver heating. Nanoparticle powder could be observed under optical microscopy (fluorescence, differential phase contrast, polarized light).

## 4. Discussion

### 4.1. In Search of the Ideal Magnetic Nanoparticle for Two-Stage Microwave Hyperthermia

With much research still ongoing, one aspect that requires clarification in the near future is the development of an ideal magnetic nanoparticle suitable for two-stage microwave hyperthermia treatment. As shown in our toxicity tests, Fe_3_O_4_ nanopowders exhibit good biocompatibility and are among the preferred particles for this technique. According to existing evidence, an “optimal” nanoparticle for two-stage microwave hyperthermia would likely have the following structural characteristics: an iron oxide core (magnetite/maghemite) or ferrite, with high saturation magnetization and controlled magnetic anisotropy [43]. In the single-domain regime, the size is approximately 15–30 nm and should be shaped to enhance anisotropy, potentially allowing alignment under the applied field to maximize heating [44]. The surface coatings should ensure biostability, low immunogenicity, uniform dispersion, and, if desired, targeting moieties or tumor-responsive shells [45]. A dose and localization strategy should ensure sufficient edema or tumor uptake to alter tissue dielectric/magnetic properties, enabling microwaves to deposit more energy locally.

Still, several aspects remain uncertain and require further study over the next few years. Firstly, there is a great need to optimize nanoparticles for microwave frequencies (GHz range) vs. classical magnetic hyperthermia (kHz-hundreds kHz), as microwave heating involves also dielectric losses, interaction with the electric field of the microwave, and often tissue heterogeneities [46]. There is a continuous trade-off between size and shape in in vivo treatments. For example, highly anisotropic or large particles may exhibit less favorable pharmacokinetics, more rapid clearance, or poorer distribution. Thermal modeling under realistic tissue conditions (blood perfusion, heterogeneous tissues, heat sinks) can reduce expected heating, so the optimal nanoparticle in vitro may underperform in vivo. Finally—and most importantly—when considering treatments in patients, safety limits on field strength, power, and tissue damage from microwave antennas, among other factors, may limit how far one can push the parameters.

### 4.2. Determining the Optimal Two-Stage Microwave Hyperthermia Protocol

Optimizing a two-stage microwave hyperthermia protocol requires integrating thermal modeling, electromagnetic field distribution, and biological response analysis. The goal is to determine the spatiotemporal heating pattern that maximizes tumor cytotoxicity while maintaining normal tissue temperatures below tolerance thresholds.

The first stage (conditioning phase) maintains tissue temperature between 40 and 42 °C for a period t1. The objectives of this stage are primarily two: to increase tumor perfusion, thereby improving oxygenation and heat transfer, and to precondition cellular metabolism to enhance sensitivity to subsequent thermal exposure [47]. Uniform heating, typically achieved through power optimization, is desired to minimize the variance of the temperature distribution. The second stage (ablative phase) elevates the temperature to 43–45 °C for a duration of t2, inducing irreversible damage. The thermal dose is quantified using the Cumulative Equivalent Minutes at 43 °C (CEM_43_), which should exceed 240 min in tumoral tissues and be less than 30 min in normal tissue [47]. Moreover, the optimal transition between stages is another relevant aspect that depends on several factors, including tumor size, dielectric heterogeneity, blood perfusion rate, and tissue thermal relaxation time. Computational modeling using the Pennes bioheat equation, combined with inverse optimization algorithms (e.g., genetic algorithms or gradient-based methods), enables the prediction of the optimal time–temperature sequence [48]. The two-stage approach balances therapeutic selectivity and treatment safety, improving both specific absorption ratio efficiency and thermal dose selectivity relative to single-phase heating. Continuous efforts should be made to optimize thermal uniformity, tumor cytotoxicity, and normal tissue protection, while incorporating electromagnetic–thermal coupling, patient-specific modeling, and adaptive feedback control, allowing precise modulation of spatial heating profiles.

From a cellular and subcellular perspective, identifying the optimal two-stage microwave hyperthermia protocol should target multiple molecules and pathways that are pathologically altered in liver tumors. According to research on other tumor types, hyperthermia exerts multifaceted biological effects on tumor cells, influencing oxidative stress, protein regulation, DNA integrity, and cellular architecture. Elevated temperatures (typically 42–45 °C) enhance reactive oxygen species (ROS) production, leading to mitochondrial dysfunction, cytochrome c release, and activation of caspase-3–mediated apoptosis [49]. These effects are accompanied by modulation of heat shock protein (HSP) pathways, particularly HSP70- and HSF1-dependent pathways, that can either promote cell survival or, under certain conditions, sensitize cells to apoptosis [50]. Hyperthermia also induces DNA damage, including telomerase inhibition, chromosomal instability, and degradation of oncogenic viral proteins such as HPV E6, thereby restoring p53-mediated apoptosis and cell-cycle arrest [51].

In addition to genotoxic and oxidative effects, hyperthermia disrupts multiple cellular structures and signaling mechanisms. It causes cytoskeletal disorganization, loss of integrin function, and microtubule collapse, contributing to detachment-induced cell death [52]. Gene expression profiling reveals broad transcriptional reprogramming, with upregulation of stress- and apoptosis-related genes (e.g., HSPA1B, DUSP1, PML) and downregulation of proliferation-associated genes (CCNE1, CEBPE) [53]. Moreover, hyperthermia can damage collagen fibers, alter cell differentiation (e.g., hemoglobin synthesis in K562 cells), and induce microvessel injury, leading to ischemic tumor necrosis [54]. Collectively, these mechanisms demonstrate that hyperthermia promotes tumor regression through coordinated oxidative, genetic, and structural disruptions within malignant tissues.

### 4.3. Translation to Clinical Research and Daily Clinical Use

Translating two-stage microwave hyperthermia using magnetic nanoparticles from preclinical research to clinical reality requires simultaneous progress on four fronts: (1) robust demonstration of biological benefit and safety, (2) patient-specific treatment planning and real-time thermometry, (3) standardized nanoparticle dosing and delivery strategies, and (4) regulatory pathways that address the combined device–drug nature of the approach. Preclinical evidence indicates that a conditioning mild-heat stage (≈40–42 °C) enhances perfusion and nanoparticle/drug accumulation, while a subsequent focused ablative stage (≈43–45 °C) triggers thermal drug release and augments cytotoxicity—mechanisms that have been validated in multiple animal models and in vitro systems employing iron-oxide magnetic nanoparticles and thermosensitive drug carriers. These mechanistic data support clinical investigation of two-stage protocols as adjuncts to locoregional therapies in hepatocellular carcinoma and liver metastases [55].

Clinical translation depends on accurate prediction and control of electromagnetic and thermal fields in the heterogeneous liver environment. The liver’s perfusion, dual blood supply, and complex dielectric properties require patient-specific electromagnetic–thermal simulations for reliable dosimetry. Modern pipelines couple full-wave electromagnetic solvers with bioheat models (Pennes or perfusion-modulated variants) to estimate CEM43 distributions and optimize antenna placement, power trajectories, and nanoparticle dosing before treatment. Such modeling has been explicitly demonstrated for interstitial microwave systems and for magnetic-fluid hyperthermia in liver geometry, and is now an expected component of trial protocols to reduce off-target heating and preserve liver function. Real-time MR thermometry or invasive fiber-optic thermocouples provide intra-procedural feedback for adaptive control of the two-stage sequence [56].

From a clinical research standpoint, early-phase trials should prioritize feasibility and safety endpoints while collecting rigorous pharmacokinetic and dosimetric data. Trials for heat-activated drug formulations (notably lyso-thermosensitive liposomal doxorubicin) and combinational hyperthermia modalities have already established applicable precedents—demonstrating the feasibility of heat-triggered drug release in liver tumors and identifying key pitfalls, such as suboptimal heating uniformity and the timing of heating relative to drug delivery, that must be addressed in MNP-assisted microwave protocols. Consequently, phase I designs should include: standardized nanoparticle characterization and dose escalation; route of administration (intra-arterial infusion vs. systemic); contemporaneous imaging of nanoparticle deposition; and careful thermal mapping during the conditioning and ablative stages to correlate local temperatures with drug release and biological response [57].

Practical workflow and device requirements must be adapted for routine oncology practice. The clinical procedure can be modularized into the following steps: (i) imaging and selection (multi-phase CT/MRI to assess tumor size, vascular anatomy and proximity to bile ducts/major vessels), (ii) nanoparticle infusion (tailored to tumor perfusion and EPR characteristics), (iii) computational planning (thermal optimization), (iv) two-stage microwave delivery under MR or probe guidance, and (v) post-treatment imaging and biomarker follow-up. Specialized interventional suites that combine microwave generators with MR-compatibility (or reliable thermometry alternatives) and user interfaces for automated power modulation based on model predictive or PID control will accelerate adoption by reducing operator variability. Multidisciplinary teams, including interventional radiology, medical physics, pharmacy/nanomedicine, and hepatology, are essential for reproducibly implementing this workflow [58].

Safety and regulatory issues related to nanoparticles are essential. Iron oxide-based nanoparticles have an established clinical footprint as MRI contrast agents, which is advantageous for rapid translational entry; however, hyperthermia applications require the additional demonstration of predictable heating behavior (specific absorption under clinical alternating magnetic fields and/or microwaves), absence of harmful aggregation, and acceptable clearance or biodegradation profiles. Aggregation or uneven intratumoral distribution can produce “hot spots” and focal overheating; therefore, quality control of particle size, coating (e.g., dextran/PEG), and magnetic/dielectric properties must be mandated in clinical protocols. Thermal safety is equally critical: the hepatic parenchyma tolerates only brief windows of supraphysiologic temperatures; therefore, adaptive control algorithms and conservative safety constraints during treatment planning are necessary to prevent biliary or vascular injury [59].

Another relevant direction is integration with other established liver cancer therapies. Two-stage hyperthermia can serve as an adjunct to transarterial chemoembolization (TACE) or percutaneous microwave ablation (MWA), or as an enhancer of thermosensitive liposomal chemotherapy; each combination has mechanistic plausibility and some clinical precedent supporting improved local control. In particular, coupling MNP-assisted heating with temperature-triggered liposomes could yield high intratumoral drug concentrations while preserving systemic exposure profiles; careful timing (drug administration shortly before or during the conditioning phase) and matching of thermal kinetics to liposome release thresholds will determine therapeutic success. Systematic trials that compare combined modalities to standard-of-care arms are required to quantify survival benefit, effects on liver function, and complication rates [60].

Finally, to accelerate and broaden adoption of the technique, there is a need to standardize outcome metrics (thermal dose maps, nanoparticle biodistribution metrics, imaging biomarkers of response), establish manufacturing pathways for clinical-grade nanoparticles, and engage regulatory agencies early to define combined device-drug evaluation frameworks.

## 5. Conclusions

Liver cancer continues to pose significant global health challenges due to its high incidence, late diagnosis, and poor therapeutic outcomes. Conventional treatment modalities, though improved through advances in surgery, chemotherapy, targeted agents, and immunotherapy, remain limited by toxicity, resistance, and suboptimal survival benefits. Two-stage microwave hyperthermia using magnetic nanoparticles offers a promising alternative by enabling precise, localized tumor heating that enhances cytotoxicity and synergizes with other treatments. Given its potential for selective tumor targeting with minimal systemic effects, we tested the technique’s efficacy in a preclinical rat liver model using various heating protocols.

According to our findings, the most appropriate exposure temperature for rat liver appears to be 42 °C, which induces vacuolar degeneration lesions at the focus level. Standard histological processing with paraffin embedding is not compatible with the fluorescence examination manner for the capture of Nile red-labeled nanoparticles, because the fluorochrome (liposoluble) is eluted during the process. The nanoparticles are not homogeneous (observed in fluorescence microscopy). The effects of thermoconditioning do not appear homogeneous across the liver mass or controllable with the methodology used to date. The level of hepatic inflammation, as indicated by increases in IL-6 and TNF-α levels, appears negligible based on results from analysis of rat liver homogenate.

Our approach warrants further investigation in clinically relevant models to validate the safety of two-stage microwave hyperthermia, optimize nanoparticle delivery, and establish its role as a viable adjunct or alternative in the management of liver and gastric malignancies.

## Data Availability

All data are available in the published manuscript.
